# A Red Cross Memorial

**Published:** 1915-07-24

**Authors:** 


### A Red Cross Memorial.

NEW CONVALESCENT HOME FOR WEST END HOSPITAL.

A new Convalescent Home, to be called the "Kitty Leigh Convalescent Home," which was opened last Saturday, has been placed at the disposal of the children of the West End Hospital and others in whom the founders may be interested. The donors are Captain and Mrs. Gerard Leigh.

The home accommodates six children, who are to be changed each month, and the nurse in charge will be Miss Mabel Thurgood, of the West End Hospital. The home is a memorial of a sister of Captain Gerard Leigh, who was engaged in Red Cross work.

